# Anti-NMDA receptor encephalitis from ovarian teratoma and mimicking psychosis: a report of a patient from a tertiary center in Pakistan

**DOI:** 10.1097/MS9.0000000000004441

**Published:** 2026-03-31

**Authors:** Muhammad Moin-Ud-Din-Arshad, Muhammad Bilal Akram, Rubab Darwesh, Jaber Hamad Jaber Amin, Muhammad Abdullah Masood, Amna Arif, Ayesha Arif, Aqsa Jamal, Laiba Fatima

**Affiliations:** a Department of Medicine, Quaid-e-Azam Medical College, Bahawalpur, Pakistan; b Department of Medicine, Fatima Jinnah Medical University, Lahore, Pakistan; c Department of Medicine, University of Sinnar, Sinnar, Sudan; d Amna Inayat Medical College, Lahore, Pakistan

**Keywords:** anti-NMDAR encephalitis, autoimmune psychosis, immunotherapy, ovarian teratoma, seizures

## Abstract

**Introduction and Importance:**

Anti-N-methyl-D-aspartate receptor (anti-NMDAR) encephalitis is a severe but treatable autoimmune neurological disorder caused by antibodies against the NMDAR, a key modulator of synaptic plasticity, memory, and behavior. Initially characterized by Dalmau *et al* in 2007, it has emerged as the most common autoimmune encephalitis, especially among young women, often associated with ovarian teratomas. Early recognition and combined immunotherapy–surgical management are essential for favorable outcomes.

**Case Presentation:**

We report a 21-year-old woman who presented with acute behavioral disturbance, hallucinations, and seizures, initially misdiagnosed as a primary psychiatric illness. Cerebrospinal fluid analysis confirmed anti-NMDAR antibodies, while brain MRI revealed left temporal hyperintensities. Initial immunotherapy produced partial improvement. Pelvic MRI subsequently detected a left ovarian teratoma, which was surgically excised. Following resection and continued corticosteroid therapy, the patient achieved full neurological and psychiatric recovery. One-month follow-up revealed complete resolution of symptoms and normal functional status.

**Clinical Discussion:**

This case illustrates the diagnostic challenges of anti-NMDAR encephalitis in resource-limited settings, where early psychiatric features may delay recognition. Our experience emphasizes the critical role of gynecologic evaluation in young women with unexplained psychosis or seizures, even without pelvic symptoms. Prompt tumor removal combined with immunotherapy ensures optimal recovery.

**Conclusion:**

Anti-NMDAR encephalitis should be a leading differential in young women with new-onset psychosis and seizures. Early CSF antibody testing and pelvic imaging are vital to diagnosis, and multidisciplinary management can yield complete recovery.

## Introduction and importance

Anti-N-methyl-D-aspartate receptor (anti-NMDAR) encephalitis, first described systematically by Dalmau *et al* in 2007^[^1^]^, is an antibody-mediated autoimmune encephalitis that primarily affects young women. The disorder arises when antibodies bind to the NR1 subunit of NMDARs, resulting in receptor internalization and disrupted glutamatergic neurotransmission^[^2^]^.

HIGHLIGHTS

- Anti-N-methyl-D-aspartate receptor (Anti-NMDAR) encephalitis is a rare autoimmune disease that often mimics psychiatric illnesses, leading to delayed diagnosis.
- This case involved a young female initially misdiagnosed with a primary psychiatric disorder, later confirmed to have anti-NMDAR encephalitis through CSF testing.
- MRI revealed temporal lobe hyperintensities, and pelvic imaging identified an ovarian teratoma, a known trigger of this autoimmune condition.
- Immunotherapy alone had limited effect, but surgical removal of the teratoma led to marked neurological and psychiatric recovery.
- The case underscores the need to include autoimmune encephalitis in differential diagnoses for new-onset psychosis and seizures in young women.

It has since become recognized as the most prevalent form of autoimmune encephalitis, with a marked female predominance (approximately 4:1)^[^3^]^. Nearly 40%–50 % of affected adult women harbor an ovarian teratoma, a benign tumor containing neural tissue expressing NMDARs, which triggers an autoimmune response^[^4,5^]^.

The clinical course often progresses through distinct stages:

1. Prodromal phase – viral-like illness with fever or headache.

2. Psychiatric phase – psychosis, agitation, or catatonia frequently mistaken for primary psychiatric disease.

3. Neurological phase – seizures, dyskinesias, autonomic instability, and altered consciousness.

4. Recovery phase – gradual improvement after treatment.

Diagnosis requires the detection of anti-NMDAR antibodies in cerebrospinal fluid (CSF), which is more sensitive than serum^[^6,7^]^. Brain MRI may appear normal or reveal temporal lobe hyperintensities. EEG may show the characteristic “extreme delta brush” pattern^[^8^]^.

Early and aggressive therapy – high-dose corticosteroids, IVIG or plasma exchange, and tumor removal when present – improves prognosis, with 75%–80 % achieving good recovery^[^9^]^. Delayed diagnosis, however, increases morbidity and mortality^[^10,11^]^.

This report describes a young woman from Pakistan whose anti-NMDAR encephalitis was initially misdiagnosed as a psychiatric disorder, highlighting diagnostic pitfalls and the life-saving impact of multidisciplinary care.

## Case presentation

A 21-year-old previously healthy female undergraduate presented with a 10-day history of severe headache followed by progressive confusion, hallucinations, and aggressive behavior over the preceding week. There was no family or personal history of psychiatric illness.

Before referral to our tertiary center, she experienced two generalized tonic–clonic seizures and received antipsychotics for a presumed acute psychosis. On arrival, she was disoriented, agitated, and had a Glasgow Coma Scale score of 10/15, with no focal neurological deficits.

Laboratory investigations showed leukocytosis (WBC 29.8 × 10^9^/L, 87 % neutrophils), hyponatremia (135 mmol/L), hypokalemia (2.7 mmol/L), and elevated CRP (7.8 mg/dL). CSF examination revealed mild lymphocytic pleocytosis (12 cells/µL), elevated protein (68 mg/dL), and positive anti-NMDAR IgG antibodies, confirming the diagnosis.

### Neuroimaging

**Figure 1.**
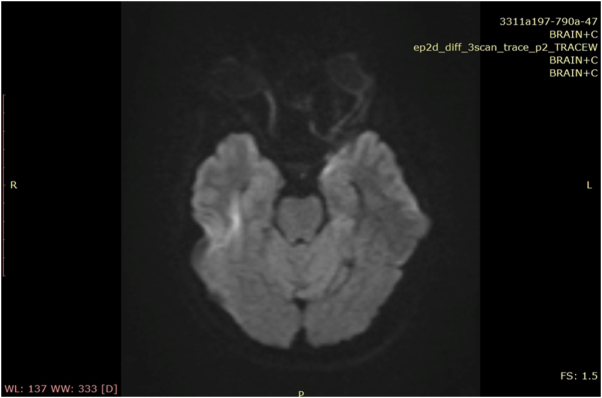
MRI brain showing hyperintensity flair in the right temporal lobe.

Brain MRI showed left temporal lobe T2-FLAIR hyperintensities (Fig. 1). EEG demonstrated diffuse slowing with intermittent rhythmic delta activity.

### Treatment course

High-dose IV methylprednisolone (1 g/day for 5 days), ceftriaxone, and acyclovir empirically were initiated.

Mild clinical improvement was noted after 1 week.

Plasmapheresis was begun but discontinued after three sessions due to a catheter-related infection.

**Figure 2.**
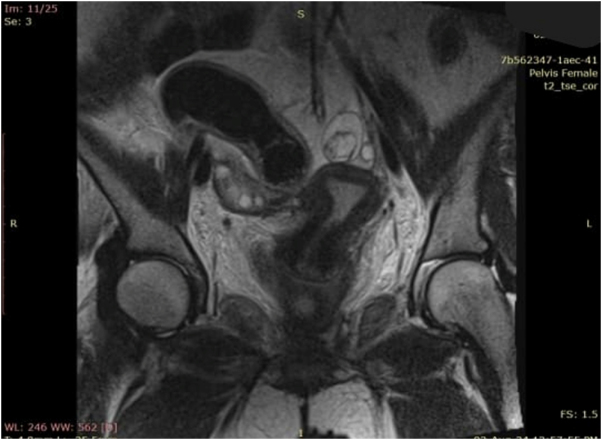
MRI pelvis showing left cystic adnexal mass consistent with an ovarian teratoma.

Pelvic MRI revealed a left ovarian teratoma (Fig. 2), which was surgically excised on hospital day 15.

- Postoperatively, the patient received another 5-day pulse of corticosteroids followed by oral prednisolone taper.

The patient’s consciousness and speech improved gradually, hallucinations resolved, and she regained full orientation by the end of the third week. She was discharged in stable condition after 28 days of hospitalization.

At 1-month follow-up, she demonstrated complete neurological and psychiatric recovery, with normal affect, memory, and gait. She had resumed academic activities and remained relapse-free. A detailed clinical chronology is summarized in Table 1.

**Table 1:** Chronological order of events.

Day	Event
Day 0	Onset of headache
Day 3	Behavioral changes
Day 7	Seizures, admission
Day 8	MRI brain, labs
Day 10	CSF + ve for anti-NMDAR antibody
Day 12	Immunotherapy started
Day 16	Pelvis MRI revealed teratoma
Day 18	Surgery
Day 22	Clinical improvement
Follow-up (1 month)	Complete recovery

## Discussion

Anti-NMDAR encephalitis exemplifies the intersection of neurology, psychiatry, and gynecology, and is frequently under-recognized in low-resource settings. Initially characterized by Dalmau *et al*^[^1^]^, it is now understood as a multistage, antibody-mediated disease targeting synaptic NMDARs^[^2^]^.

Our patient’s presentation – rapid onset of psychosis, seizures, and autonomic instability – mirrored the classical disease pattern^[^3,4^]^. However, like many cases, she was initially misdiagnosed with a primary psychiatric disorder, delaying definitive therapy. Such diagnostic pitfalls remain a major concern, particularly in young women^[^5,6^]^.

### Diagnostic considerations

The positive CSF anti-NMDAR antibodies confirmed the diagnosis. MRI findings of temporal hyperintensities and EEG slowing further supported limbic involvement^[^7,8,12^]^. CSF antibody testing remains the gold standard due to its higher sensitivity compared to serum assays^[^6^]^.

### Therapeutic approach

First-line therapy (steroids, IVIG, plasmapheresis) aims to suppress antibody production and inflammation^[^9^]^. Our patient’s partial response to initial immunotherapy improved markedly after surgical resection of the ovarian teratoma, consistent with evidence that tumor removal accelerates recovery and reduces relapse^[^11,13^]^.

### Prognosis and recovery

Early recognition and comprehensive management yield favorable outcomes in up to 80% of patients^[^9^]^. In our case, the patient’s full recovery within 1 month underscores the reversibility of this disorder when promptly treated.

### Learning points/novel contribution

- The case underscores the diagnostic challenge of distinguishing autoimmune psychosis from primary psychiatric illness, particularly in regions where antibody testing is limited.
- It highlights the role of gynecologists – any woman of reproductive age presenting with new-onset psychosis or seizures warrants pelvic imaging to exclude teratoma^[^4,5^]^.
- It demonstrates the effectiveness of multidisciplinary collaboration between psychiatry, neurology, and gynecology in achieving full recovery.

## Conclusion

When young women present with acute psychosis, seizures, or encephalopathy, clinicians should consider anti-NMDAR encephalitis early. Prompt CSF antibody testing and pelvic imaging are crucial. Combined immunotherapy and surgical tumor removal remain the cornerstone of treatment. Early multidisciplinary intervention dramatically improves outcomes, as shown in our patient who achieved complete recovery within 1 month.

## Data Availability

Not applicable.

## Research registration unique identifying number (UIN)

Not applicable – registration is not required for case reports.

## Peer and provenance

Not commissioned; externally peer-reviewed.
